# Indirect Evolution of Hybrid Lethality Due to Linkage with Selected Locus in *Mimulus guttatus*

**DOI:** 10.1371/journal.pbio.1001497

**Published:** 2013-02-26

**Authors:** Kevin M. Wright, Deborah Lloyd, David B. Lowry, Mark R. Macnair, John H. Willis

**Affiliations:** 1 Department of Biology, Duke University, Durham, North Carolina, United States of America; 2 Department of Organismic and Evolutionary Biology, Harvard University, Cambridge, Massachusetts, United States of America; 3 School of Biosciences, Hatherly Laboratories, University of Exeter, Exeter, United Kingdom; 4 School of Biological Sciences, The University of Texas at Austin, Austin, Texas, United States of America; Institute of Science and Technology Austria (IST Austria), United Kingdom

## Abstract

Most species are superbly and intricately adapted to the environments in which they live. Adaptive evolution by natural selection is the primary force shaping biological diversity. Differences between closely related species in ecologically selected characters such as habitat preference, reproductive timing, courtship behavior, or pollinator attraction may prevent interbreeding in nature, causing reproductive isolation. But does ecological adaptation cause reproductive incompatibilities such as hybrid sterility or lethality? Although several genes causing hybrid incompatibilities have been identified, there is intense debate over whether the genes that contribute to ecological adaptations also cause hybrid incompatibilities. Thirty years ago, a genetic study of local adaptation to copper mine soils in the wildflower *Mimulus guttatus* identified a locus that appeared to cause copper tolerance and hybrid lethality in crosses to other populations. But do copper tolerance and hybrid lethality have the same molecular genetic basis? Here we show, using high-resolution genome mapping, that copper tolerance and hybrid lethality are not caused by the same gene but are in fact separately controlled by two tightly linked loci. We further show that selection on the copper tolerance locus indirectly caused the hybrid incompatibility allele to go to high frequency in the copper mine population because of hitchhiking. Our results provide a new twist on Darwin's original supposition that hybrid incompatibilities evolve as an incidental by-product of ordinary adaptation to the environment.

## Abstract 2

Ecological selection on an adaptive allele causes a tightly linked hybrid incompatibility factor to rapidly hitchhike to high frequency in a population of the wildflower *Mimulus guttatus*.

## Author Summary

Adaptive evolution by natural selection is the primary force generating biological diversity. A critical question is whether the evolution of hybrid incompatibility, which is essential for the maintenance of species diversity, is caused by adaptive evolution. In this article, we investigate one of the most widely cited examples of ecological divergence driving the evolution of reproductive incompatibility, the strong association between hybrid lethality and copper tolerance in a copper mine population of the wildflower *Mimulus guttatus*. Hybrid lethality and tolerance of high levels of copper co-segregate as a single Mendelian locus. While copper tolerance and hybrid lethality are nearly universal in the mine population at Copperopolis, California, they are absent from adjacent off-mine populations, suggesting that reproductive isolation evolved rapidly as a pleiotropic by-product of recent adaptation to the mine environment. We find that copper tolerance and hybrid lethality are controlled by distinct loci, in tight genetic linkage. We also demonstrate that this genomic region has experienced strong recent selection and conclude that ecological selection for copper tolerance indirectly caused the neighboring hybrid lethality allele to hitchhike to high frequency. To our knowledge, this is the first case to demonstrate that reproductive isolation factors can evolve as an incidental by-product of adaptation to novel environments through genetic hitchhiking.

## Introduction

Adaptation to local environmental conditions by natural selection is the primary cause of evolutionary change in natural populations. Ecological adaptation can cause reproductive isolation when selection acts on traits that influence the likelihood of intermating in nature, such as habitat preference, reproductive timing, courtship behavior, or pollinator attraction [1],[2]. However, it is controversial whether adaptation to local environmental conditions promotes the development of reproductive incompatibilities such as hybrid sterility or lethality. As Darwin [3] first discussed, reproductive incompatibility should not evolve directly via natural selection (but see [4]) and would only arise as an incidental by-product of interspecific divergence. Darwin realized that reproductive isolation could evolve if it is not expressed as lineages diverge, perhaps due to populations inhabiting geographically distinct regions, and it would only manifest in cases in which these populations hybridized subsequent to divergence. Classic models of the evolution of hybrid incompatibility, independently developed by Bateson [5], Dobzhansky [6], and Muller [7] (BDM), predict that alleles at different loci may accumulate within distinct lineages, and although they may be neutral or adaptive in an ancestral population, they will produce deleterious interactions when brought together by hybridization. This model is now thoroughly supported by genetic mapping studies of hybrid incompatibility loci. The role of natural selection in driving the evolution of incompatibility alleles continues to be an area of rich investigation [8]–[10].

Selection may drive the evolution of BDM incompatibility alleles in a variety of ways. With the molecular genetic dissection of multiple hybrid incompatibility systems, researchers have determined that many of the underlying loci have experienced rapid evolutionary divergence consistent with natural selection [9],[10], but there is little evidence of what may be causing this rapid divergence. Evidence for the traditional view that hybrid incompatibility evolves as a by-product of adaptation to divergent environments is supported by only a few lab-based experimental evolution studies [11]–[13]. Of the hybrid incompatibility genes identified in natural populations, it seems unlikely they function in classical ecological adaptation. Instead researchers have speculated that these incompatibilities evolved due to intragenomic conflict [14],[15]. For example, the incompatibility *Overdrive* locus causes both segregation distortion and hybrid male sterility in *Drosophila psuedoobscura* [16]. Ultimately, it is considerably difficult to determine the evolutionary forces acting upon hybrid incompatibility alleles during the process of divergence because these studies focus on species reproductively isolated for thousands of generations.

In this study, we investigate whether recent adaptation to a copper mine habitat by the wild flower *Mimulus guttatus* caused the evolution of hybrid incompatibility. Extreme soil environments, either naturally occurring or created anthropogenically, characterized by low nutrient and high heavy-metal concentrations, impose strong selection on plant communities [17],[18]. *M. guttatus* has colonized multiple mine sites in the Sierra Nevada foothills region of central California, the largest of which is located at the Keystone Union mine complex in the town of Copperopolis [19]. A survey of tolerance to high copper concentration in plants from Copperopolis and adjacent off-mine populations suggests this phenotype is under strong selection in the mine habitat. Copper tolerance is nearly fixed in the mine population (99.77%, *N* = 2,796) and segregating at very low frequency in a sample of 15 off-mine populations located with 40 km of Copperopolis (8.7%, *N* = 1,440) [20]. An investigation into the genetic basis of this trait initiated over 30 years ago identified a locus that appeared to cause both copper tolerance and hybrid lethality in crosses to other off-mine populations [21],[22]. Copper tolerance is controlled by a dominant allele at a single Mendelian locus, when measured as a threshold character in lab-based root growth assays. Crosses between tolerant lines from a recombinant backcross population (Figure S1) and the off-mine population, Cerig-y-drudion, Wales, United Kingdom (hereafter referred to as *Cerig*), yielded various levels of F1 hybrid lethality, whereas crosses between nontolerant lines from the same recombinant backcross population always produced viable offspring [22]. Additional crosses demonstrated there is a single incompatibility locus in the Copperopolis population and that variation in F1 hybrid lethality is caused by multiple loci segregating in the *Cerig* population [22]. *Cerig* was used in the initial crosses by M. R. Macnair and continues to be a focal population in this study, because it was the first population identified to produce inviable hybrid offspring in crosses with Copperopolis [22]. Subsequent experiments have demonstrated that the *Cerig* hybrid lethality factors are geographically widespread and segregate in three California populations. In contrast, the Copperopolis lethality factor is geographically restricted; no incompatible plants were identified in test crosses with plants from 15 other populations (*N* = 10 plants/population) [23]. The lethality factor is at high frequency in the Copperopolis population; 16 Copperopolis genotypes (five of which are from this study, see details below) have been tested and all of them produce lethal offspring when crossed to *Cerig* tester lines [22],[23]. Hybrid lethality in this system is consistent with the BDM model and was not observed to be genetically de-coupled from the copper tolerance phenotype [22],[24]. One attractive hypothesis is that they are controlled by the same locus and F1 hybrid lethality evolved as a pleiotropic by-product of the locally adaptive copper tolerance. An alternative hypothesis is that the two phenotypes are not pleiotropic and are instead controlled by two distinct loci in tight genetic linkage.

The physiological manifestation of BDM hybrid incompatibility in this system reveals possible genetic mechanism for inviability, while maintaining support for both the pleiotropy and linkage hypotheses. Hybrid lethality manifests in F1 plants as tissue yellowing and death in the early stages of development and prevents the plants from reaching reproductive maturity [22],[24]. This lethality is similar to the hybrid necrosis phenotype known for many years in the plant agriculture literature [25]. Recently, it has been demonstrated that hybrid necrosis is an autoimmune-like response due to negative interactions between divergent pathogen-resistance proteins, often involving nucleotide-binding leucine-rich repeats (NB-LRRs) and their interacting partners [26],[27]. Although there is no support for NB-LRR's dual function in disease resistance and trafficking of heavy-metal ions, this could be the case for their partner proteins. NB-LRR protein–protein interactions are difficult to examine, and most partners remain unknown [28]. Additionally, there is strong evidence of pleiotropy between disease resistance and heavy metal tolerance from an independent class of pathogen-resistance proteins, defensins, which contribute to enhanced zinc tolerance in *Arabidopsis halleri* [29]. It remains to be determined whether selection on the copper tolerance allele in *M. guttatus* resulted in the near fixation of a hybrid incompatibility factor because these phenotypes are the result of pleiotropy at a single locus.

The primary aim of this study is to investigate the genetic basis of the copper tolerance and hybrid necrosis phenotypes to determine whether they are pleiotropic or controlled by distinct genes in tight genetic linkage. We also use population genetic approaches to infer the nature of natural selection acting on this copper tolerance and hybrid necrosis genomic region.

## Results

### Genetic Mapping

**Figure 1:**
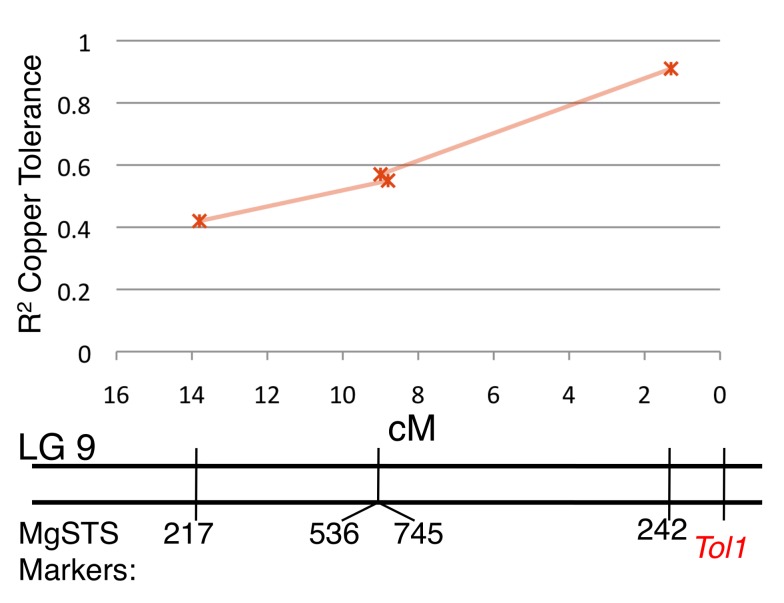
Markers linked to *Tol1*. Association between four LG9 markers with copper tolerance phenotype.

To determine whether copper tolerance and hybrid lethality are controlled by the same locus, we conducted a high-resolution genetic mapping experiment. Our mapping approach focused on first identifying the location of copper tolerance locus because, of the two phenotypes, it is easier to measure, and then screen a subset of plants with recombination events near the tolerance locus for hybrid inviability. In order to genetically map these phenotypes with high resolution, we used a near isogenic line (NIL) created through recurrent backcrossing of a Copperopolis by *Cerig35* F1 (this line is compatible with Copperopolis) to lines derived from Stinson Beach, California. For seven generations, only tolerant plants were selected to be backcrossed to Stinson Beach (Figure S1). The Stinson Beach population is nontolerant and lacks any intrinsic incompatibilities with Copperopolis [23]. We used multiple outbred Stinson Beach lines to avoid the development of inbreeding depression in our backcross lines. Each backcross generation segregated tolerant and nontolerant progeny in equal ratio, in accordance with predictions for a single, dominant tolerance locus. We created our mapping population by crossing a single tolerant (BC_7T_) and a single nontolerant (BC_7NT_) line to produce F1_BC7_ progeny (Figure S1). We set about identifying the introgressed tolerance region in the NIL lines (BC_7T_, BC_7NT_) by screening 541 genetic markers distributed across the genome (mimulusevolution.org). We identified 42 markers that were homozygous in the BC_7NT_ line and heterozygous in the BC_7T_ line, possibly indicating a region of introgression from the tolerant parent. To establish which heterozygous markers are linked to the copper tolerance locus, we tested for an association between phenotype and genotype in 80 F1_BC7_ lines. We measured copper tolerance in the F1_BC7_ plants as a threshold character in accordance with previously published hydroponic methods [20]. We found the genotypes of one marker, *MgSTS242*, located on linkage group (LG) 9, were very tightly correlated with tolerance phenotype (Figure 1). We henceforth refer to this copper tolerance locus as *Tol1*.

**Figure 2:**
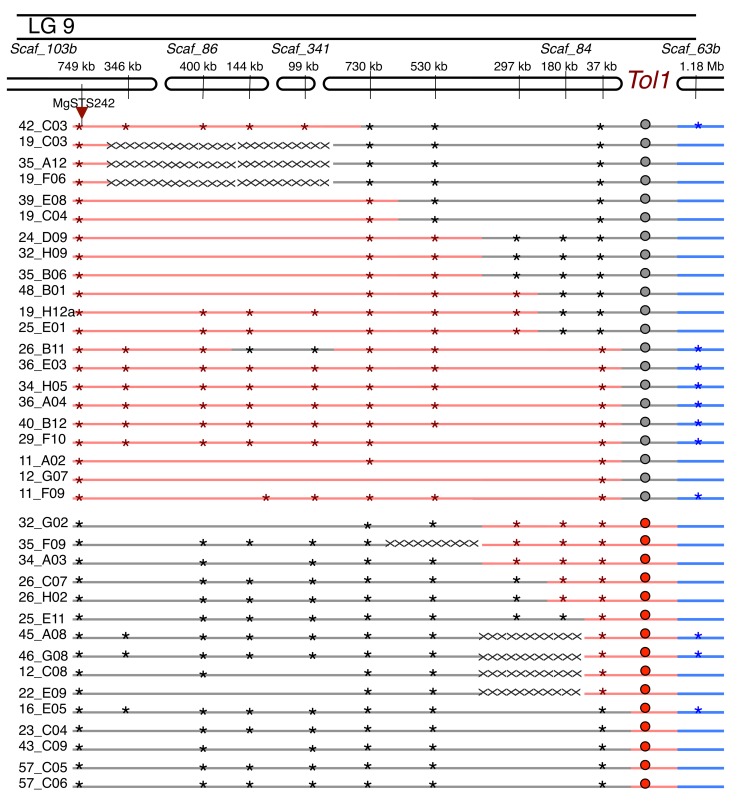
Fine scale genetic map of *Tol1*. Fine mapping of *Tol1*. Asterisks indicate individual marker genotypes. Lines denote imputed genotypic states for each individual. Red is introgressed region, heterozygous for Copperopolis allele, and black is homozygous for Stinson Beach allele. Blue indicates region is outside of introgressed segment. Cross-hatches indicate regions of unknown genotype. Phenotypic state is shown in circles; red is tolerant and gray is nontolerant.

To fine map the genomic position of *Tol1*, we created a mapping population of 4,340 F1_BC7_ plants, phenotyped each plant for copper tolerance, and genotyped each plant with the *MgSTS242* marker. Initially, we identified 83 recombinant plants, however we repeatedly phenotyped and genotyped these lines and we were able to confirm 36 true recombinant plants, indicating that this marker is 0.83 cM from the *Tol1* (Figure 2). To fine map *Tol1*, we designed additional genetic markers near *MgSTS242* using the currently unpublished *M. guttatus* genome assembly (www.phytozome.net). This assembly is not contiguous on the scale of chromosomes; the genome is instead composed of many, relatively small, genomic scaffolds that do not assemble into large chromosome-length blocks. The assembly in the *Tol1* genomic region is particularly poor because this is a highly repetitive pericentromic region of LG 9 and none of the scaffolds that map to this region in the reference are longer than 1 MB. We attempted to determine the location of 10 target scaffolds relative to *MgSTS242* and *Tol1* by designing multiple new markers per scaffold and screening them in our recombinant lines. We identified three scaffolds that are in tight linkage to *Tol1* (Figure 2). We determined that a marker on scaffold 84 is in tightest association with *Tol1*; we found 14 plants have a recombination event between *Sc84_37kb* and *Tol1*, indicating this marker is located within 0.32 cM of the tolerance locus (Figure 2). We identified three additional scaffolds that map to this region (97c, 157, and 238), but they do not reside within the *Sc84_37kb*–*Tol1* interval (Text S1). We have not included these scaffolds in our map because we have a limited amount of genetic material and we could not test all 36 recombinants. We attempted to identify a flanking marker for *Tol1* by designing markers in the 3′ end of scaffold 63b, which is predicted to be adjacent to the 5′ end of scaffold 84 (unpublished data, Uffe Hellsten, Joint Genome Institute, Walnut Creek, CA). However, we found no evidence of linkage between our markers and the tolerant phenotype, likely indicating that scaffold 63b is outside of the NIL introgression region or there are errors in the reference genome assembly (Figure 2 and Text S1). We observed the same pattern of no linkage between *Tol1* and markers located in two additional scaffolds (460 and 925) that are predicted to be located in this region in the reference genome assembly.

**Figure 3:**
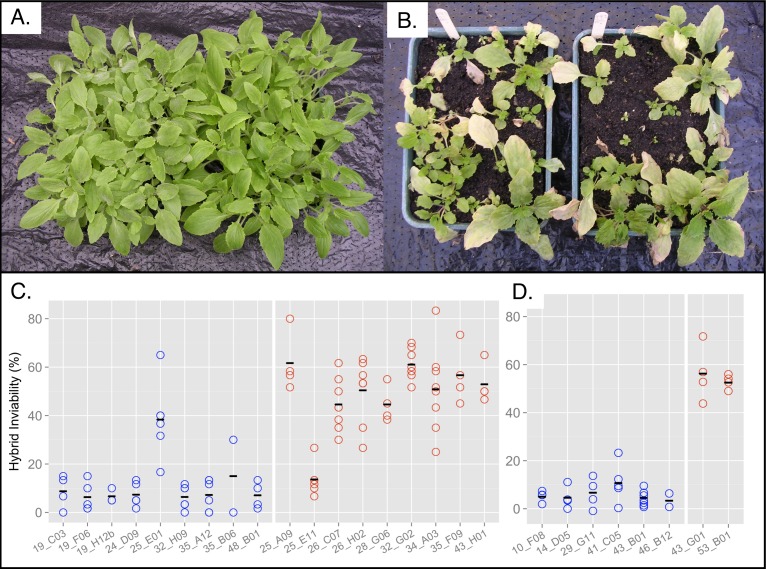
Hybrid lethality of recombinant lines. Photos of healthy (A) and lethal (B) progeny grown under identical greenhouse conditions. The percentage of inviable offspring derived from crosses between F1_BC7_ lines and *Cerig10*. Recombinant F1_BC7_ lines (C) have recombination breakpoints between MgSTS242 genetic marker and *Tol1*. Control F1_BC7_ lines (D) do not have recombination breakpoints between MgSTS242 and *Tol1*. Points are replicate measurements of the number of inviable offspring in a block of 60 seedlings. Blue points denote nontolerant lines and red points denote copper tolerant lines.

In order to determine if the tolerance and incompatibility phenotypes are controlled by the same locus, we crossed 18 (nine tolerant and nine nontolerant) recombinant plants to an incompatible genotype, *Cerig10* (Figure S2). Crosses were conducted in each direction to determine if there is asymmetry in the incompatibility. We measured hybrid inviability as the percentage of offspring with a majority of tissue being yellow or necrotic in a block of 60 plants (Figure 3A and 3B). We scored hybrid inviability in 2–8 replicate blocks per line. We used *Cerig10* as our tester line because this line produced the highest level of hybrid lethality in crosses to Copperopolis lines. In crosses between *Cerig10* and five Copperopolis genotypes, we found an average of 83% of the offspring were inviable (Figure S2).

Our F1_BC7_×*Cerig10* crosses produced a bimodal distribution of hybrid inviability. The majority of crosses with nontolerant lines produced 5%–15% inviable progeny, whereas the majority of crosses with tolerant lines produced 45%–62% inviable progeny (Figure 3C and 3D). We identified two plants, 25_E01 and 25_E11, with recombinant phenotypes (Figure 3C). Hybrid inviability of nontolerant line 25_E01 is significantly elevated compared to all nontolerant control lines (Wilcoxon test, *z* = 2.46, *p*<0.014; Table S1) except 46_B12, which had only two replicate measurements (Wilcoxon test, *z* = 1.84, *p* = 0.065; Table S1). Hybrid inviability of tolerant line 25_E11 is significantly lower than the two tolerant control lines (Wilcoxon test, *z* = 2.46, *p*<0.014; Table S1). This establishes that copper tolerance and hybrid inviability are controlled by two distinct, but tightly linked, loci and that hybrid necrosis is not a pleiotropic by-product of copper tolerance. We henceforth refer to the hybrid inviability locus as *Nec1*.

**Figure 4:**
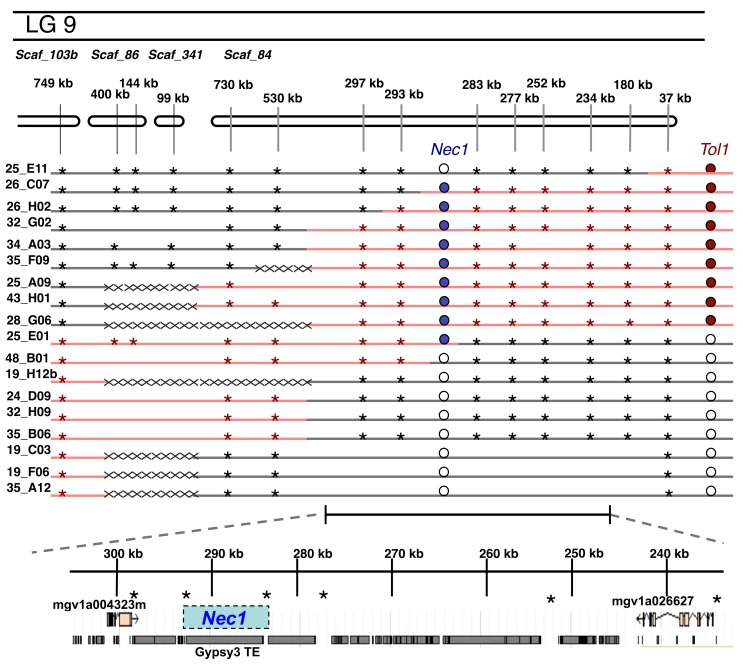
Genetic map of *Nec1* region. Symbols are the same as described in Figure 2. Annotation of *Nec1* region shows predicted structure of two genes flanking *Nec1*, mgv1a004323m and mgv1a026627. The repetitive elements in this region are shown as grey blocks below genes. *Nec1* is almost entirely restricted to a Gypsy3 TE. Annotation based on *M. guttatus* Annotation v1.1, phytozome.net.

To map the genomic location of *Nec1*, we compared the *Tol1* genotyping information for these lines to our lethality data and determined that *Nec1* maps to scaffold 84. To fine map *Nec1*, we designed markers in this region and genotyped plants with informative recombination events. We found that the incompatibility locus maps to an intergenic region between 283 kb and 293 kb on scaffold 84 (Figure 4). This region in the *M. guttatus* genome (www.phytozome.net) contains a single *Gypsy3* transposable element (TE) (Figure 4). We are prevented from continuing to narrow this region, despite identifying three lines (26_C07, 25_E01, and 48_B01) with recombination events within this locus (Figure 4) because of the repetitive nature of this element. We attempted to sequence across this genomic interval using long-range PCR, but these attempts were unsuccessful. Our long-range PCR may have failed because repetitive DNA in this TE element or because this genomic interval is much larger in the Copperopolis genome compared to the reference genome.

*Nec1* maps to an intergenic region containing a *Gypsy3* TE. Unregulated TE replication can cause hybrid sterility in *Drosophila*; in this system, hybrid sterility manifests in crosses in a single direction because hybrids lack the maternally inherited TE replication-suppression mechanisms [30]. It is unlikely that this is the cause of inviability in our system because significant levels of hybrid lethality occur in both crossing directions, although it is higher when *Cerig* is the paternal parent (Table S2). Instead, the functional changes at *Nec1* likely result from altered gene expression or gene function.

The Copperopolis allele at *Nec1* may cause hybrid lethality by altering the *cis*-regulatory expression of a gene within this region, and the functional gene may flank our mapped interval or reside even further away along the chromosome. The nearest gene, MGV1A004323M, resides 5 kb from *Nec1* and encodes a glycosyltransferase metabolic enzyme (www.phytozome.net). This protein has greatest homology to *A. thaliana* gene AT3G18170, which attaches glyosyl groups to N-linked glycan molecules critical for construction of plant cell membranes [31]. The second flanking gene, MGV1A026627M, is located 41 kb from *Nec1* and encodes a Jumonji-C histone demethylation protein. The Jumonji-C proteins are part of the histone demethylase family, whose function reverses the epigenetic silencing of transposons and genes in eukaryotic genomes [32]. Neither of these genes, nor is any gene within 100 kb of Nec1, is a known participant in plant disease-resistance pathway. Alternatively, *Nec1* may involve another gene that is absent from the reference genome. This gene may have undergone recent functional or expression changes to cause negative interactions with a member of the plant disease-resistance pathway, producing the hybrid necrosis phenotype. This hypothesis is especially attractive because the NB-LRRs often exhibit large variation in copy number within species [33]. Although additional work is required to distinguish between the possible mechanisms of *Nec1* function, our results clearly demonstrate that hybrid inviability did not evolve as a pleiotropic by-product of the evolution of copper tolerance. What evolutionary force caused the lethality allele at *Nec1* to go to high frequency in the Copperopolis population?

### Selection Driving the Evolution of Hybrid Incompatibility

The hybrid lethality phenotype reached high frequency in the Copperopolis population because of genetic drift or natural selection. Genetic drift may have been the primary evolutionary mechanism causing the lethality allele at *Nec1* to rise to high frequency if the initial population of mine colonists experienced a bottleneck event. This scenario would cause a genome-wide reduction in genetic variation in the mine population relative to neighboring off-mine populations [34]. Alternatively, the hybrid lethality allele at *Nec1* may have been directly selected because this allele confers some fitness advantage in the mine habitat. A variant of the selection hypothesis is that the lethality allele at *Nec1* may have hitchhiked to high frequency because of selection on the tightly linked *Tol1* locus. In this case, the tolerance and incompatibility alleles would have resided on the same haplotype in the founders of the mine population. Selection on this genomic region would leave a molecular genetic signature of reduced genetic variation in the mine population and increased genetic differentiation between mine and off-mine populations in this genomic region, compared to the rest of the genome [35]–[38].

Studies that attempt to identify these molecular signatures of selection in natural populations have been used to identify putatively selected loci in many different systems [39]–[44]. The power of population genetic studies to detect signatures of selection can be diminished by multiple interdependent processes: selection on standing genetic variation [45], population structure [46], and time since a selective sweep [34]. These studies can most readily identify a molecular signature of selection in the classic case of a hard sweep, in which strong selection on a new mutation causes a single haplotype to rapidly go to fixation and tightly linked alleles hitchhike to high frequency [34]–[38]. However, if this mutation is segregating in the ancestral population and has recombined onto multiple haplotypes prior to selection, hitchhiking of tightly linked alleles is reduced [45]. This event, termed a “soft sweep,” will not decrease genetic variation or increase population differentiation at linked alleles to the same degree as a hard sweep. The number of generations since a selected allele goes to high frequency affects the molecular signature of selection because each generation there is new opportunity for mutation, migration, and recombination to add genetic variation to the derived population [34]. The young age of the mine habitat (150 y) suggests that if a sweep occurred, the molecular signature of this event would still be apparent. The mine and off-mine populations are in close geographic proximity, and there is opportunity for gene flow, suggesting there will be little effect of population structure. Gene flow will increase the amount of shared genetic variation at selectively neutral sites in the genome, but strong selection in the mine environment could maintain the original haplotype (or haplotypes) containing the tolerance allele in the face of gene flow.

In order to evaluate the selection hypothesis, we compared genetic variation at markers linked to *Tol1* and markers randomly distributed throughout the genome. We sampled individual plants, collected as seed from unique maternal families in 2005 and 2007, from the mine population at Copperopolis (*N* = 108) and from two off-mine populations: O'Byrnes Ferry Road (*N* = 33), located 2 km from Copperopolis, and Hunt Road (*N* = 39), located 9 km from Copperopolis (Figure S3). The geographic proximity of these populations and recent divergence of the mine population, a maximum of 150 generations, suggests any signal of selection will not be lost in a haze of population structure. Furthermore, this population sampling is designed to explicitly test for an effect of habitat-mediated selection on a single genomic region with known phenotypic effect, *Tol1*/*Nec1* loci, and not a genome-wide analysis. Lastly, this design will not be confounded by the effect of hybrid inviability because these off-mine populations lack the incompatible allele found in the *Cerig* population [23].

We measured genetic variation of eight loci in the *Tol1* fine mapped region (*Tol1_Link_*) and 11 unlinked loci (*Tol1_UnLink_*) for the Copperopolis and two off-mine populations. The *Tol1_UnLink_* markers serve as a control to estimate the average level of genetic variation and population differentiation. We used co-dominant DNA fragment length polymorphism markers [47] to estimate the within-population genetic variation, which we report as number of alleles (*N_a_*) and expected heterozygosity (*H_e_*) [48]. We report both values, but these are often correlated when using fragment length markers. We calculated genetic differentiation between populations as *F_st_* for two marker classes: *Tol1_Link_* and *Tol1_UnLink_* using *Fdist* [36] implemented in the program LOSITAN [49]. To evaluate whether *F_st_* is significantly elevated in the *Tol1* fine mapped region, we compared the observed levels of *F_st_* at the *Tol1_Link_* markers to a null distribution of *F_st_* generated from coalescence simulations of population divergence using the *Tol1_UnLink_* dataset in the program LOSITAN. Our hypothesis is that *F_st_* for the *Tol1_Link_* markers will be significantly higher than the *Tol1_UnLink_* markers. The coalescence simulations assume two populations and use Wright's symmetrical Island Model of migration [50] to calculate the probability that two randomly chosen chromosomes in a population have a most recent common ancestor within that population without an intervening migration or mutation event [36],[49]. This model also assumes that the two populations are at equilibrium with constant effective population size. Our populations likely violate this assumption because the mine population was only recently established. However, Beaumont and Nichols found that a recent colonization event had no significant effect on their estimates of the *F_st_* distribution [36].

We observe low *F_st_* for all *Tol1_UnLink_* markers, and for all but a single marker, there is no significant differentiation for the three pairwise population comparisons (Table S3). Using the *Tol1_UnLink_* dataset, we also estimate the relatedness between the three populations using the program STRUCTURE version 2.3 [51]. In this analysis, the two off-mine populations cluster more tightly with each other than with Copperopolis (Figure S4); thus, we combined the two off-mine populations into a single sample for subsequent analyses.

**Figure 5:**
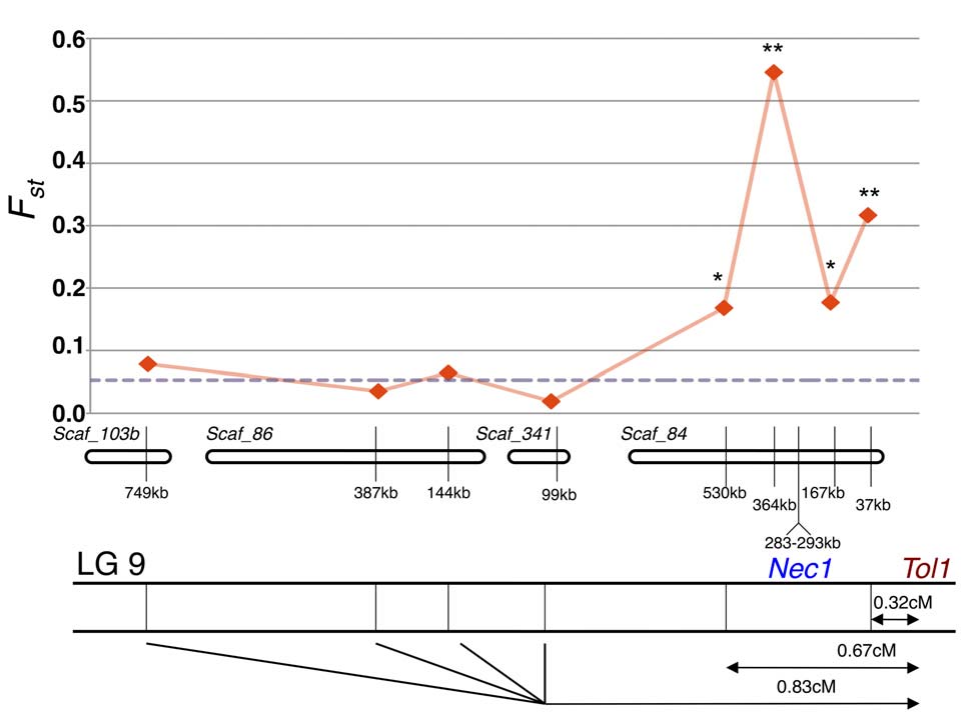
*F_st_* of *Tol1_Link_* markers. *F_st_* values for *Tol1_Link_* markers distributed along LG 9. Dashed line is mean *F_st_* for *Tol1_UnLink_* dataset. * *p*<0.025. ** *p*<0.0025.

We found mine and off-mine populations have no difference in *N_a_* and only slightly reduced *H_e_* for the *Tol1_UnLink_* markers (Table 1). This suggests that the mine population did not undergo a dramatic population bottleneck during colonization, or if it did, this signal has been erased by ongoing gene flow. Conversely, we find that *N_a_* and *H_e_* of the *Tol1_Link_* markers is sharply reduced in the Copperopolis population compared to the off-mine population, and this pattern is strongest at the four markers in tightest linkage to *Tol1* and *Nec1* (Table 1). We found that the four markers that are most tightly linked to *Tol1* and *Nec1* have significantly elevated *F_st_* when compared to the *Tol1_UnLink_* distribution (Figures 5 and S5). *F_st_* values varied between the four markers; the markers with the two highest values are *Sc84_364kb* (*F_st_* = 0.546) and *Sc84_37kb* (*F_st_* = 0.317) are in tightest linkage to the *Nec1* and *Tol1* loci, respectively. These data support the two predictions arising from the selection hypothesis: low genetic variation within Copperopolis and high differentiation between populations at markers linked to the tolerance and hybrid lethality loci.

**Table 1:** Genetic variation of *Tol1_Link_* and *Tol1_UnLink_* markers.

Locus	Dist from *Tol1* (cM)	N Off	N Mine	N_a_ Off	N_a_ Mine	H_e_ Off	H_e_ Mine
Tol_UnLink_	50	130–144	210–216	15.5 (6.71)	15.1 (6.67)	0.815 (0.19)	0.773 (0.19)
Tol_Link_	0.32–0.83	122–142	204–216	10.1 (4.85)	5.50 (2.82)	0.727 (0.25)	0.446 (0.27)
Sc103_749kb	0.83	140	216	13	9	0.841	0.684
Sc86_144kb	0.83	126	204	11	7	0.848	0.738
Sc86_387kb	0.83	124	206	4	2	0.139	0.010
Sc341_99kb	0.83	122	208	6	7	0.712	0.689
Sc84_530kb	0.67	134	214	16	9	0.880	0.501
Sc84_364kb	∼0.46	136	206	7	3	0.716	0.121
Sc84_167kb	∼0.35	126	216	17	4	0.866	0.479
Sc84_37kb	0.32	142	216	7	3	0.813	0.349

N is number of chromosomes sampled, N_a_ is number unique alleles, and H_e_ is the estimated heterozygosity. Mean and standard deviation for N_a_ and H_e_ for each marker class are shown.

## Discussion

### Genetic Dissection of Copper Tolerance and Hybrid Inviability

We demonstrate that copper tolerance is not pleiotropic with hybrid inviability. Instead, both phenotypes are controlled by distinct loci that are in tight genetic linkage. We genetically mapped the copper tolerance locus to a highly repetitive pericentromeric genomic region. The large amount of repetitive DNA and relatively rare euchromatic sequence has inhibited the assembly large genomic scaffolds in this region of *M. guttatus* reference genome (unpublished data, Uffe Hellsten, Joint Genome Institute, Walnut Creek, CA). Our attempts to fine map the *Tol1* locus to a genomic scaffold, or even identify a flanking marker, have been stymied by the complex nature of this genomic region despite our large mapping population. In contrast, we were able to fine map the hybrid lethality locus to a 10 kb intergenic region with a contiguous genomic scaffold. The Copperopolis allele at *Nec1* may cause hybrid lethality by altering the *cis*-regulatory expression of a neighboring gene, or this region in the Copperopolis genome may harbor genes that are missing in the reference *M. guttatus* genome. We attempted to amplify the 10 kb region from the Copperopolis genome using long-range PCR, but these attempts were unsuccessful. The failed PCR attempts may be caused by repetitive DNA within the gypsy-3 TE or because this region may be expanded or rearranged in the Copperopolis genome compared to our reference.

We observed a large amount of variance between independent replicates of the high-lethality lines (Figures 3C, 3D, and S2). This variance is likely caused by two factors—segregating variation at multiple incompatibility loci in our tester line, *Cerig10*, and variation in temperature in the greenhouses in which the F1_BC7_×*Cerig10* offspring were grown. There are multiple incompatibility loci segregating within the *Cerig* population that interact with the Copperopolis allele at the locus we have now identified as *Nec1* [22],[23]. Our tester line, *Cerig10*, was chosen because it gave the highest level of incompatibility, but it may be heterozygous at some incompatibility loci, inflating variation in hybrid lethality. An additional source of variation may be the temperature at which the offspring were grown; research on hybrid necrosis in *A. thaliana* has demonstrated that this phenotype manifests at 16°C but is absent at 23°C [27]. Our plants were grown under controlled greenhouse conditions, but daily or seasonal temperature fluctuations may have exceeded the capability of the greenhouse to maintain constant temperature.

### Selection for Copper Tolerances Drives Evolution of Hybrid Incompatibility

We find strong support for the hypothesis that adaptation to the mine habitat imposed selection on the *Nec1*/*Tol1* genomic region in the Copperopolis population. The high level of genetic variation in the *Tol1_UnLink_* dataset in the Copperopolis population argues against a dramatic bottleneck event during mine colonization. The four markers in tight linkage to *Tol1* and *Nec1* have reduced *N_a_* and *H_e_* in the mine population, as well as significantly elevated *F_st_*. We interpret these data as evidence of a sweep at *Tol1*, driven by selection for copper tolerance, causing the *Nec1* BDM incompatibility allele to hitchhike to high frequency in the mine population. This model predicts that the marker in tightest linkage to *Tol1*, *Sc84_37kb*, would have the highest *F_st_* and sharpest reduction in *H_e_*. Contrary to this prediction, we found that marker *Sc84_364kb*, the marker nearest *Nec1*, has the highest *F_st_* and sharpest reduction in *H_e_*. This finding suggests that adaptation to the mine environment was more complex than the classic hard selective sweep at *Tol1*. Although we cannot completely rule out the possibility that the incompatibility allele at *Nec1* has also experienced selection in the mine population, the physiological effect of the tolerance allele and the biogeographic distribution of copper tolerant plants, which are nearly fixed in multiple mine populations and are segregating at 8.3% of neighboring off-mine populations, is strong evidence that copper tolerance is under selection in the mine habitat [21]. Because the lethality allele at *Nec1* has only been found in a single, very recently derived population [23] and has no discernible phenotypic effect beyond causing hybrid lethality, it is less likely this allele is experiencing selection in the mine environment.

We suggest multiple factors that may explain why the patterns of *H_e_* and *F_st_* we observe do not conform to the predictions of a classic hard selective sweep. Variation in the mutation rate between markers *Sc84_37kb* and *Sc84_364kb* may explain the differences in *H_e_* and *F_st_*. Additionally, we note that we have been unable to fine map the precise location of *Tol1* because this is a highly repetitive pericentromeric region and the *Sc84_367kb* marker may actually be closer to *Tol1* in the Copperopolis genome than its position in the reference genome suggests. Finally, we suggest that the tolerance allele at *Tol1* may not have experienced a hard selective sweep. If the tolerance allele was segregating at low frequency in the ancestral population and experienced a soft selective sweep, tightly linked sites would be affected differently depending on the amount of genetic variation at these sites in the ancestral population and the level of linkage disequilibrium (LD) between the selected allele and tightly linked sites when selection first acted upon this population [45]. Tolerant plants are segregating at 8.3% in off-mine populations [23], and although we are unable to determine whether this is caused by segregating ancestral variation or recent migration from mine to off-mine populations, it indicates that the tolerant allele may predate the development of the copper mines and recently rose to near fixation via a soft selective sweep. We investigate whether a soft sweep at *Tol1* could have caused the lethality allele at *Nec1* to hitchhike to high frequency using a quantitative model of genetic hitchhiking.

To simulate hitchhiking on tightly linked sites following a hard or soft selective sweep, we use the two-locus model of genetic hitchhiking described by Maynard Smith and Haigh [35] and Barton [38]. In the classic model of a hard selective sweep, the beneficial allele resides on a single haplotype and the selected and linked alleles are initially in complete LD. Because copper mining was initiated in the 1860s, we focused on strong selection situations that would cause a beneficial allele to become nearly fixed within 150 generations. We model the effects of hitchhiking on neutral allele segregating at low frequency (5%) in the ancestral population because the hybrid lethality allele has not been found in neighboring off-mine populations [23]. Additional details of our hitchhiking model are provided in Text S1. Consistent with previous findings [38], we find that during a hard selective sweep, tightly linked alleles within 0.5 cM hitchhike to high frequency in the derived population (Figure S6A, S6D, and S6G). We next model the soft sweep scenario, in which the selected allele is segregating at low frequency (5% and 10%) in the ancestral population. We find that a soft selective sweep can cause sites within 0.5 cM of the beneficial allele to hitchhike to high frequency for a wide range of parameter values (Figure S6B, S6C, S6E, S6F, S6H, and S6I). The primary determinant of hitchhiking effects is the initial level of LD between the selected and neutral alleles. In the hard sweep model, LD is 1, however this parameter can vary when the focal beneficial mutation and a tightly linked allele are segregating in the ancestral population. We find that when we set initial levels of LD to high levels (0.8–1.0), a tightly linked allele within 0.5 cM can hitchhike to high frequency in the derived population (Figure S6). Overall, these findings demonstrate that for this situation, in which a *M. guttatus* population has rapidly adapted to the copper mine environment, a soft or hard selective sweep can produce large hitchhiking effects on tightly linked sites that happen to be in strong LD in the ancestral population. Although this hitchhiking model makes assumptions that are likely violated in natural populations—deterministic selection on an allele, bi-allelic loci, and constant population size—it provides a useful quantitative framework to describe the effects of genetic hitchhiking on loci in tight genetic linkage.

The variation we observe in of *H_e_* and *F_st_* between *Tol1_Link_* markers suggests that adaptation in the mine environment may have occurred in a manner more complex than the classic hard sweep model. A soft selective sweep may not uniformly reduce genetic variation or increase genetic differentiation at tightly linked sites if there were differences in the amount of ancestral genetic variation at these sites or variation in the levels of LD between the selected allele and linked sites in the ancestral population [45]. In a soft sweep, the primary determinant of the strength of hitchhiking effects is the initial level of LD between the selected allele and tightly linked alleles. Unfortunately, the extent of LD in the ancestral population for this pericentromeric region is not known. Although we cannot conclusively state whether there was a hard or soft sweep at the *Tol1*/*Nec1* region, we do find strong evidence of selection on this region in the mine population, and the results of our hitchhiking simulations of a hard or soft sweep are consistent with the hypothesis that selection at *Tol1* caused the hybrid lethality allele at *Nec1* to hitchhike to high frequency in mine population.

In summary, we have determined that the copper tolerance and hybrid inviability phenotypes are controlled by two distinct, tightly linked loci. Hybrid inviability is not a pleiotropic by-product of adaptation to the mine environment, as originally proposed [22]. We find that *Tol1* maps to a highly repetitive, unassembled region of the *M. guttatus* genome, whereas *Nec1* maps to a 10 kb region containing a single TE. Furthermore, we demonstrate that there has been strong positive selection on the *Nec1/Tol1* genomic region. We interpret these data as evidence that the *Nec1* lethality allele rose to high frequency because of genetic drift induced by selection on the tightly linked *Tol1*. We demonstrate that natural selection on one locus can cause a tightly linked hybrid lethality allele to hitchhike to high frequency, providing empirical evidence for a new twist on Darwin's hypothesis that reproductive isolation can evolve as an incidental by-product of adaptation to novel environments.

## Materials and Methods

### Phenotyping

Our mapping population was created with repeated backcrossing and phenotypic selection for copper tolerance [20],[21]. The initial cross between Copperopolis and Cerig was conducted in 1981, but after the discovery of hybrid inviability loci segregating in this population, all subsequent crosses were made to the Stinson Beach population. The *Tol1*/*Nec1* mapping population was grown and maintained at the University of Exeter greenhouse from 2005–2009. Tolerance was scored as a threshold character as previously described [20]. Tolerance was measured repeatedly (2–6 times) for every putative recombinant line, although a few lines did perish in the greenhouse before they could be re-tested. To score hybrid lethality, we grew 60 seedlings from a focal cross and scored the number of plants with majority yellow or necrotic tissue after 3 wk of growth [22]. Hybrid lethality was scored in multiple (2–8) growouts per line from 2008–2009.

### Genetic Dissection of Tol1 and Nec1

Genetic mapping of *Tol1* and *Nec1* was conducted at Duke University using tissue samples shipped from University of Exeter. Genomic DNA was extracted from plant tissue using a modified hexadecyl trimethyl-ammonium bromide chloroform extraction protocol [52]. Most of the genetic markers used in this analysis are DNA fragment-length polymorphism markers analyzed using capillary electrophoresis on an ABI 3730×l DNA Analyzer [53]. These genetic markers score variation in PCR fragment length created by insertion-deletions. For most markers, primers reside in conserved exon sequence and amplify intron sequence [47]. The size of the amplified fragments was scored automatically by the program GENEMARKER (SoftGenetics, 2005, State College, PA) and was confirmed by eye. For additional fine-mapping markers, we used di-deoxy sequencing of PCR products to identify SNPs distinguishing Copperopolis and Stinson Beach alleles (Markers: *Sc84_180kb*, *234kb*, *252kb*, *277kb*, *281kb*, *283kb*, *293kb*, *297kb*; *Sc86_144kb*, *400kb*; *Sc341_99kb*). SNPs were scored manually using Sequencher (Gene Codes Corp., Ann Arbor, MI). To be sure of genotypes for markers defining the *Nec1* locus, Sc84_283kb, 293kb, and 297kb, we ran 2–3 independent reactions for each genetic line.

### Population Genetic Analyses

We sampled Copperopolis, O'Byrnes Ferry Road, and Hunt Road populations in 2005 and 2007. We collected seed from unique maternal plants and grew a single plant from each maternal line at Duke University greenhouse for genetic testing. We measured genetic variation using fragment-length polymorphism markers [47]. We calculated the number of alleles and heterozygosity using the program Arlequin [48]. We calculated observed levels of *F_st_* for *Tol1_Link_* and *Tol1_UnLink_* markers and generated null distribution of *F_st_* using *Tol1_UnLink_* markers using coalescence simulations implemented in the program LOSITAN [49] with the following settings: two populations in the Island Model, 1,000,000 simulations, neutral mean *F_st_*, and infinite allele mutation model as suggested for microsatellite markers [34]. To conduct numerical simulations of the two-locus hitchhiking model based on Maynard Smith and Haigh [35] and Barton [38], we wrote a program in C. Constant variables in each simulation were the population size, *N*, set at 1,000, and the allele frequency of tightly linked neutral allele, U_0_, set at 0.05. We ran simulations varying the strength of selection, initial frequency of selected allele, and LD between the selected allele and tightly linked sites (Figure S6).

## Supporting Information

Figure S1 Crosses to generate mapping population. The initial cross was between Copperopolis and the compatible *Cerig35* genotype. Subsequent backcrosses were conducted for seven generations to different, outbred Stinson Beach lines in each generation.
(PDF)

Figure S2 *Nec1* mapping crosses. Control and test crosses for mapping Nec1. Each individual point is the frequency of lethal offspring in a block of 60; each bar is the mean for replicate blocks within a cross. The CopxCerig control crosses were conducted with five different Copperopolis genotypes and two *Cerig* genotypes. We observed no significant difference between individual Copperopolis genotypes; thus, we present pooled data from these crosses. The second set of control crosses was between *Cerig10* or *Cerig35* and control tolerant and nontolerant F1_BC7_ lines that have no evidence of a recombination event near *Tol1*.
(PDF)

Figure S3 *M. guttatus* population map. Map highlighting three populations used in analysis of population differentiation: stars mark the location at Copperopolis, O'Byrnes Ferry Road, and Hunt Road. Red shaded region is the approximate outline of North Union–Keystone Union mine complex at Copperopolis, California.
(PDF)

Figure S4 Structure of mine and off-mine populations. Representative STRUCTURE output for k = 2 and k = 3 taken from one of three replicate runs with burnin = 100,000 and simulations = 1,000,000. There were no differences in grouping of individuals between replicate runs for k = 2 or 3.
(PDF)

Figure S5 *F_st_* marker values. Observed value of *F_st_*, scaled by *H_e_*, for *Tol1_Link_* (red dots) and *Tol1_UnLink_* (blue dots). Lines show expected null model generated from *Tol1_UnLink_* markers: dark blue, 0.9975%; light blue, 0.975%; gray, median; light purple, 0.025%.
(PDF)

Figure S6 Hitchhiking effects for linked loci. Plot of recombination frequency versus change in frequency of neutral allele, ΔU. Initial frequency of neutral allele, U_0_, set at 0.05, and the maximum ΔU is 0.95. Population size, *N*, is assumed to be constant and set at 1,000. Initial frequency of selected locus, p_0_, varies with columns (1/2*N* = 0.0005, 0.05, 0.1) and strength of selection, s, varies with row (0.05, 0.1, 0.25). *T_fix_* is number of generations at which *p*>0.99. (A, G, H) Simulations of a hard selective sweep assume new mutation, *p*, initially resides on the same haplotype as a linked allele, *u*. Overlapping lines demonstrates that our numerical model (orange) matches Barton's analytical solution (green) (equation 1 in [38]). (B, C, E, F, H, I) Soft sweep simulations assume p_0_ is 0.05 or 0.10. Different colored lines denote simulations initiated with different values of *u_p*, the frequency of the *u* allele on the *p* haplotype. orange *u_p* = 1.0; dark red *u_p* = 0.9; tan *u_p* = 0.8; dark green *u_p* = 0.7; blue *u_p* = 0.6; purple *u_p* = 0.5.
(PDF)

Table S1 Variation in level of hybrid inviability for lines 25E01 and 25E11. We tested for differences in the level of hybrid inviabilty between 25E01 or 25E11 and all other lines using the nonparametric Wilcoxon/Kruskal–Wallis test because the data are not normally distributed.
(DOCX)

Table S2 Reciprocal hybrid inviability. The observed and expected number of inviable offspring from reciprocal crosses between Cerig and *Tol1* recombinant plants. ** *p*<0.01.
(DOCX)

Table S3 Pairwise population differentiation. Pairwise population estimates of *F_st_* and *H_e_* values for *Tol1_UnLink_* markers. * *p*<0.025.
(DOCX)

Text S1 We provide details on genotyping the mapping population, identification of recombinant lines, and attempts to map additional scaffolds from the *M. guttatus* genome assembly to the Tol1 genomic region. Second, we provide additional information on our model of genetic hitchhiking under scenarios of a hard or soft selective sweep.
(DOCX)

## Abbreviations

BDM: Bateson, Dobzhanksy, Muller
cM: centimorgan
LD: linkage disequilibrium
NB-LRR: nucleotide-binding leucine-rich repeat
NIL: near isogenic line
TE: transposable element
