# Supplementary figures and images for: Indirect Evolution of Hybrid Lethality Due to Linkage with Selected Locus in Mimulus guttatus
Source: PLoS Biol. 2013 Feb 26;11(2):e1001497. doi: 10.1371/journal.pbio.1001497 (PMC3582499; doi:10.1371/journal.pbio.1001497)

## Creation of Mapping Population

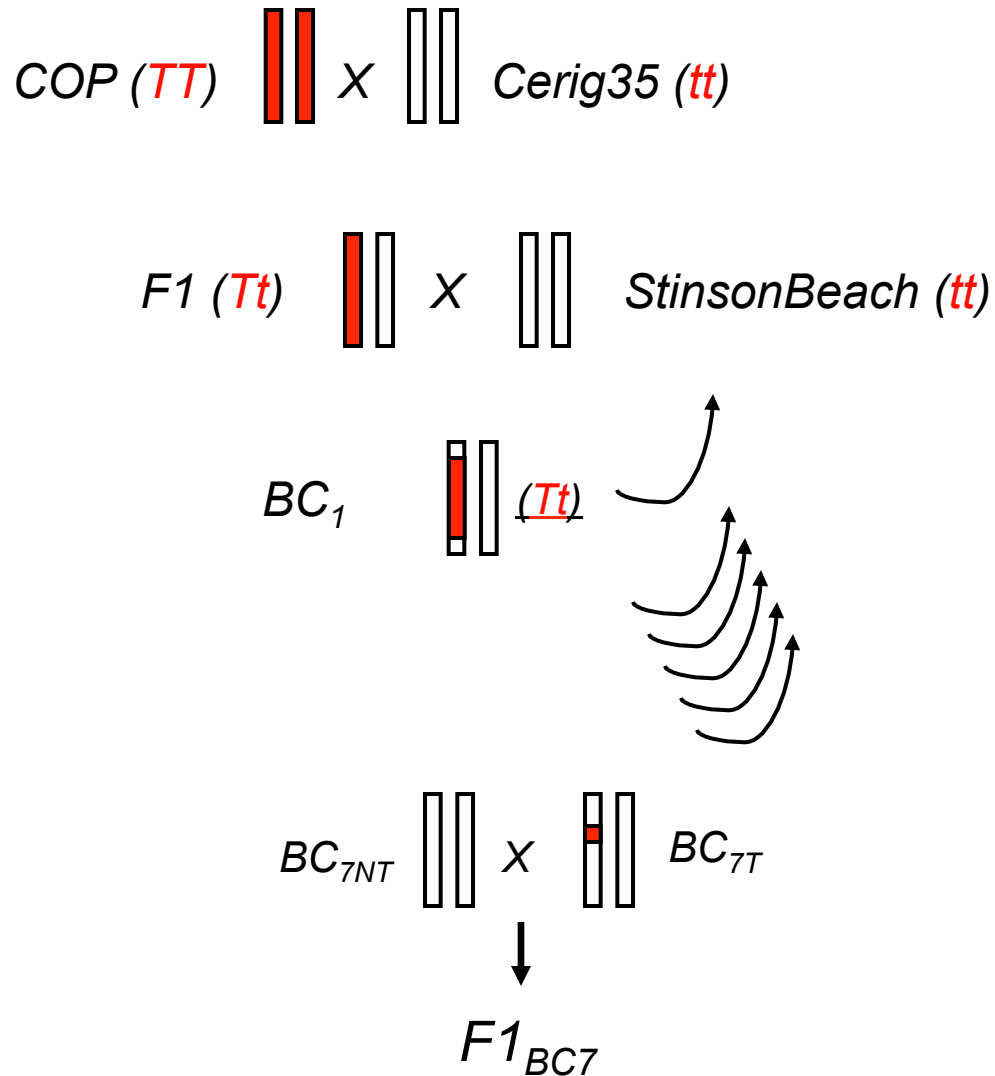

## *To1* Mapping procedure

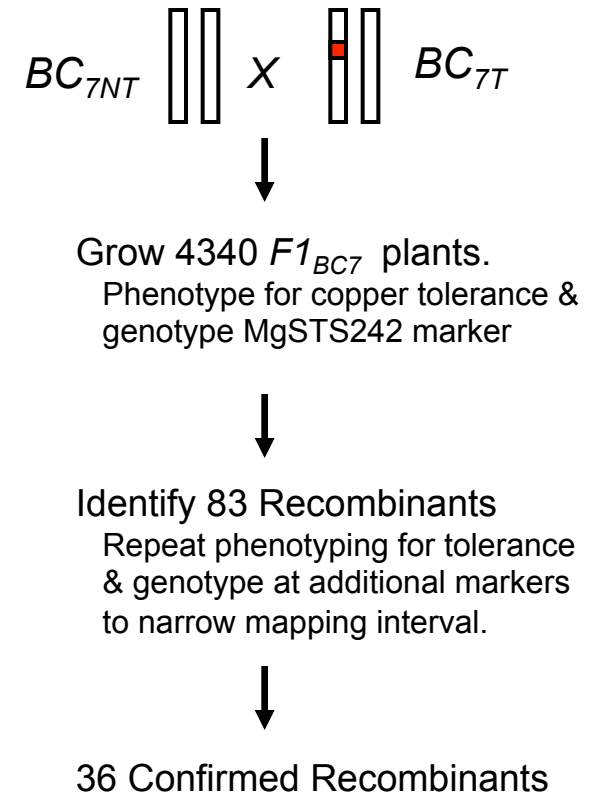

Supplement: Figure S1 — Crosses to generate mapping population. The initial cross was between Copperopolis and the compatible Cerig35 genotype. Subsequent backcrosses were conducted for seven generations to different, outbred Stinson Beach lines in each generation. (PDF) [file pbio.1001497.s001.pdf]

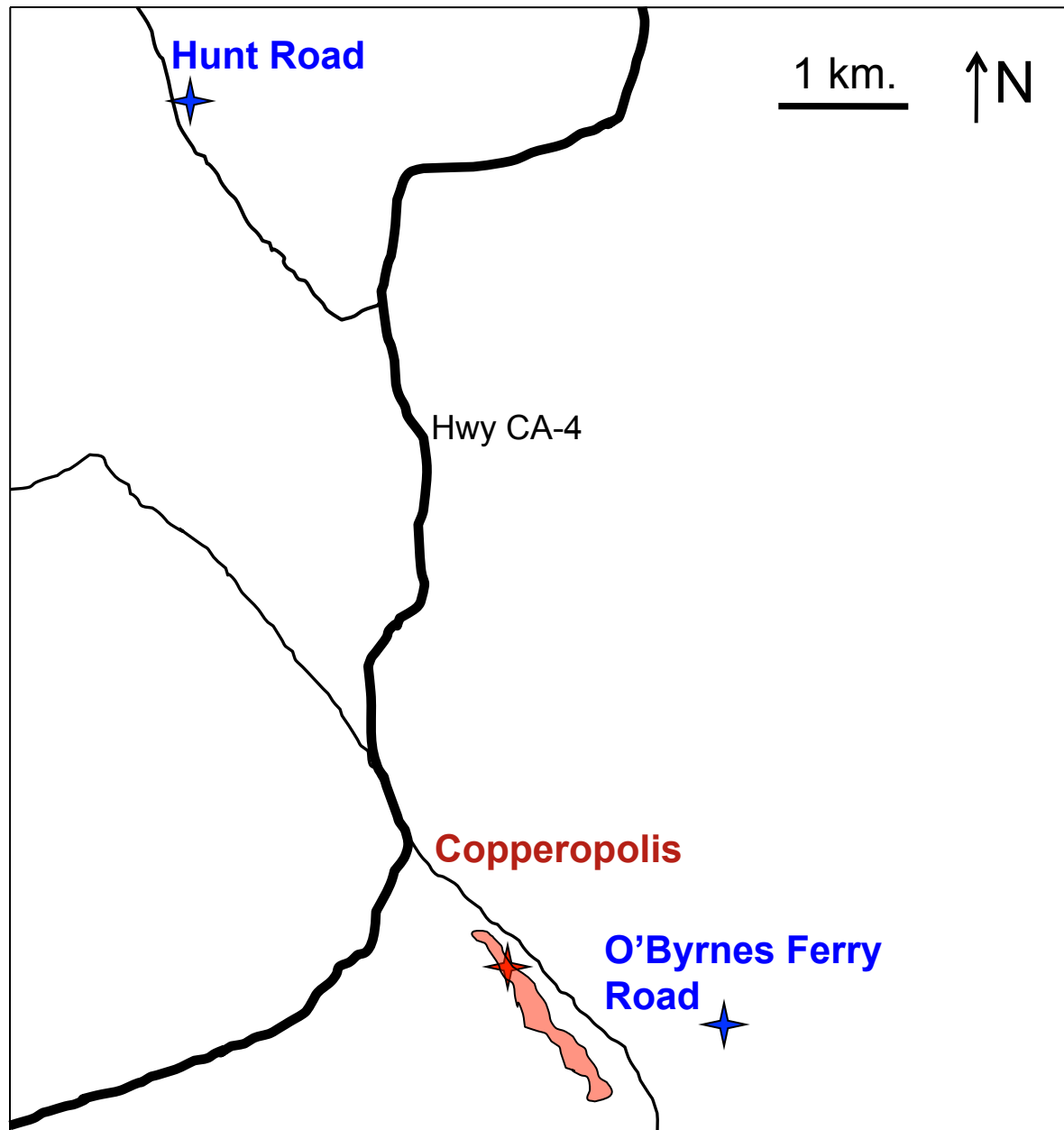

Supplement: Figure S3 — M. guttatus population map. Map highlighting three populations used in analysis of population differentiation: stars mark the location at Copperopolis, O'Byrnes Ferry Road, and Hunt Road. Red shaded region is the approximate outline of North Union–Keystone Union mine complex at Copperopolis, California. (PDF) [file pbio.1001497.s003.pdf]

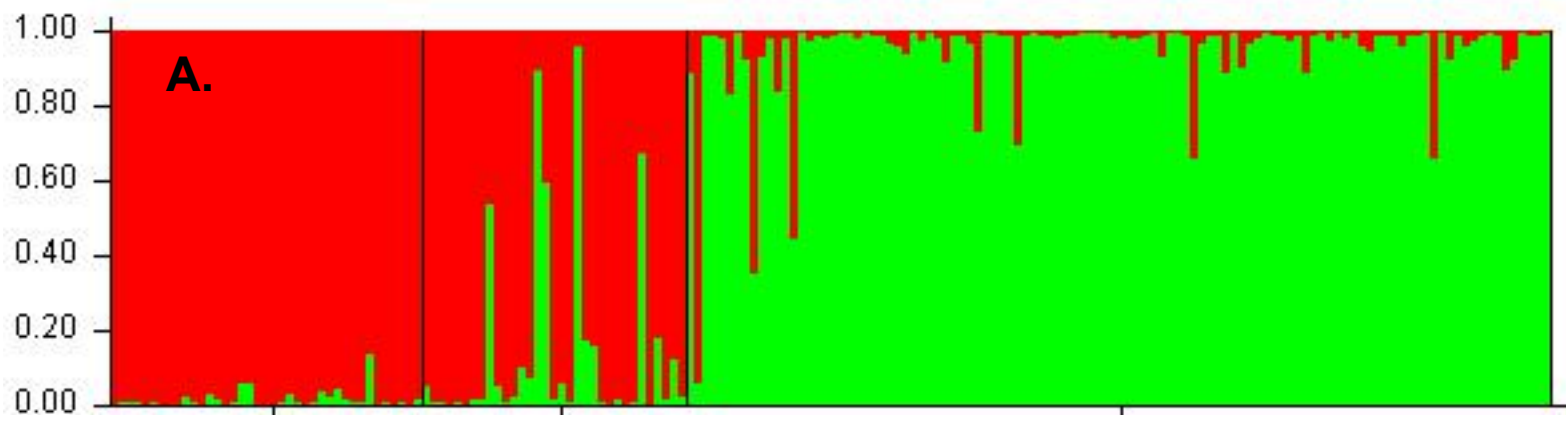

*Hunt Rd*

*O'Byrens  
Ferry Rd*

*Copperopolis*

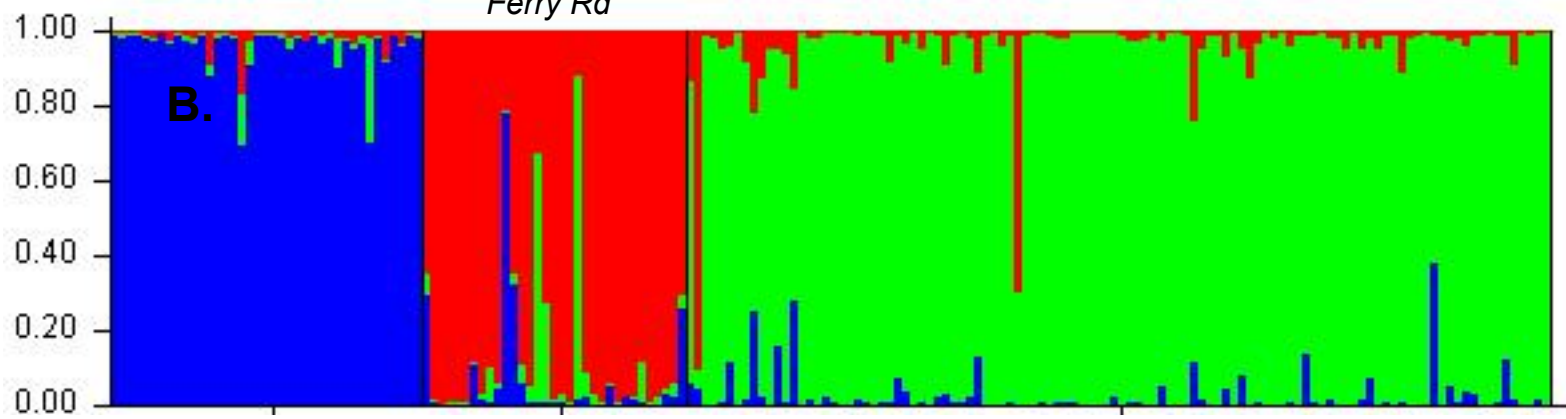

Supplement: Figure S4 — Structure of mine and off-mine populations. Representative STRUCTURE output for k = 2 and k = 3 taken from one of three replicate runs with burnin = 100,000 and simulations = 1,000,000. There were no differences in grouping of individuals between replicate runs for k = 2 or 3. (PDF) [file pbio.1001497.s004.pdf]

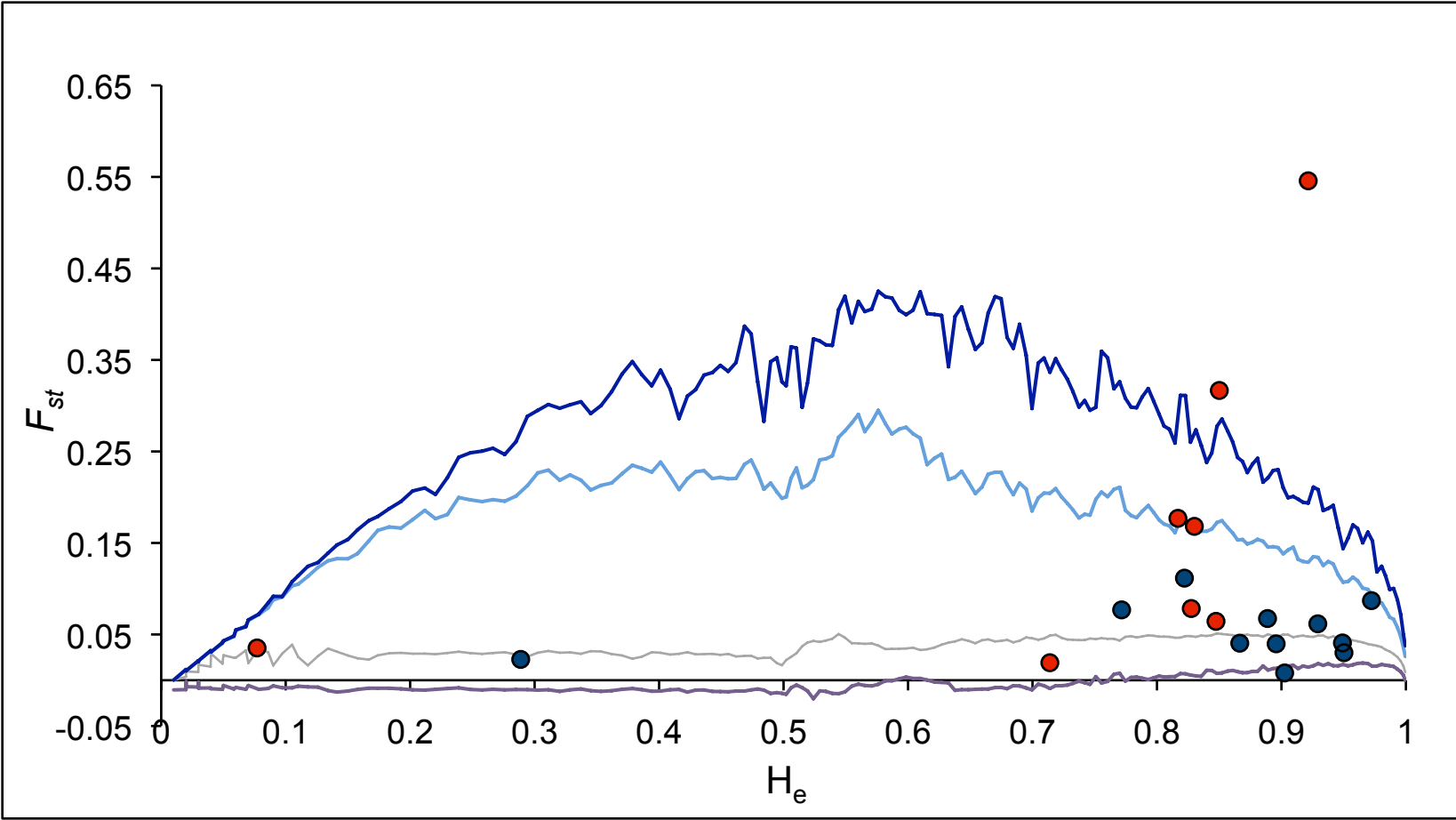

Supplement: Figure S5 — Fst marker values. Observed value of Fst, scaled by He, for Tol1Link (red dots) and Tol1UnLink (blue dots). Lines show expected null model generated from Tol1UnLink markers: dark blue, 0.9975%; light blue, 0.975%; gray, median; light purple, 0.025%. (PDF) [file pbio.1001497.s005.pdf]

$$p_o = 1/2N$$

$$p_o = 0.05$$

$$p_o = 0.10$$

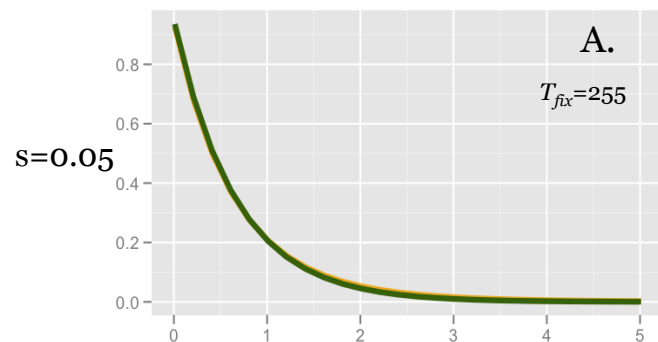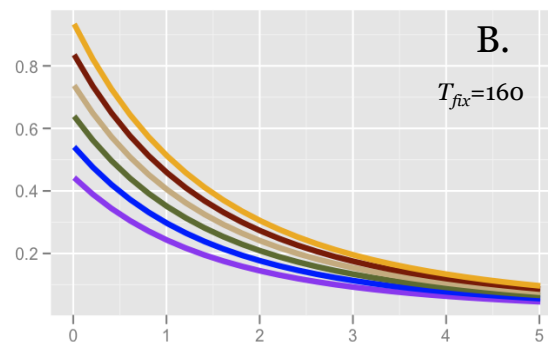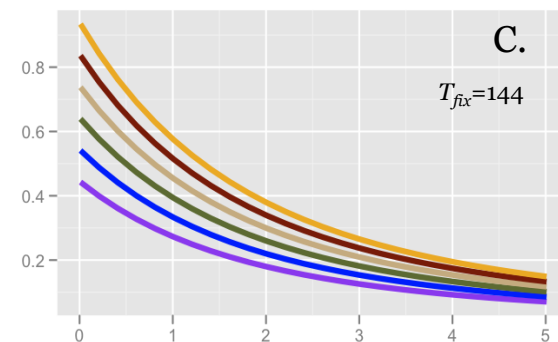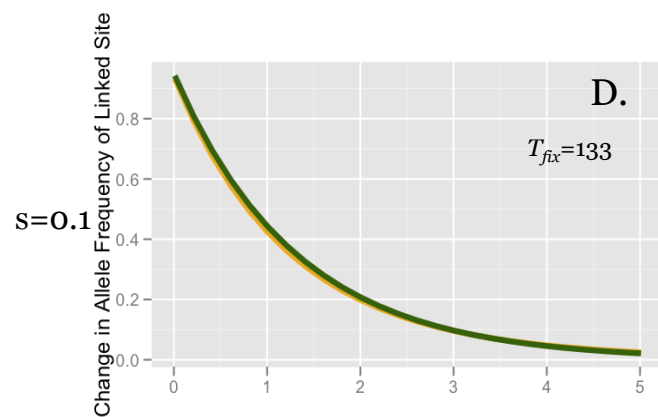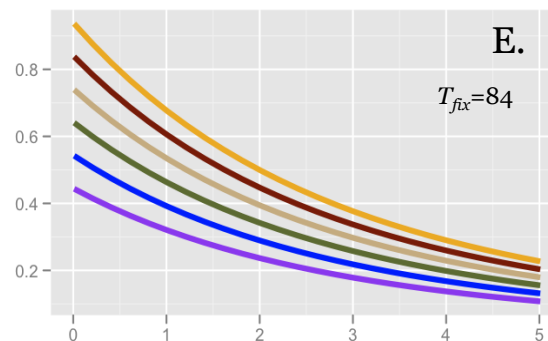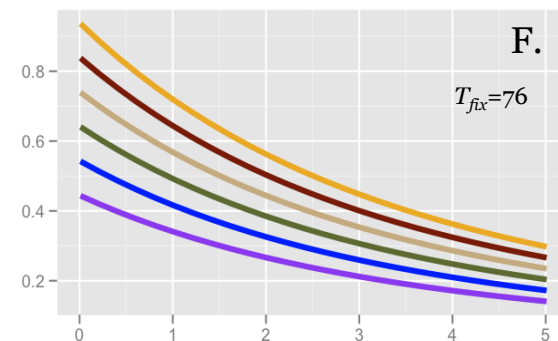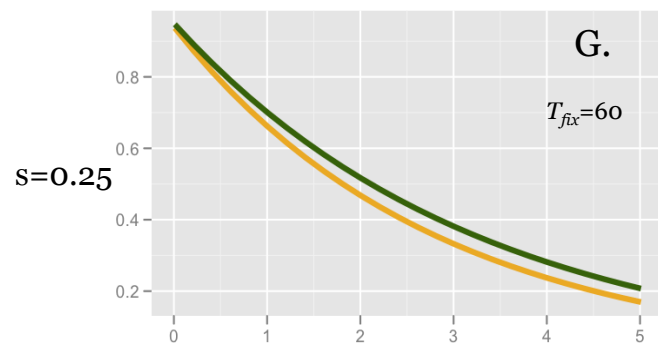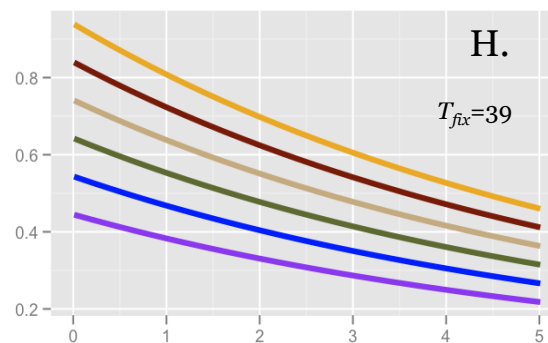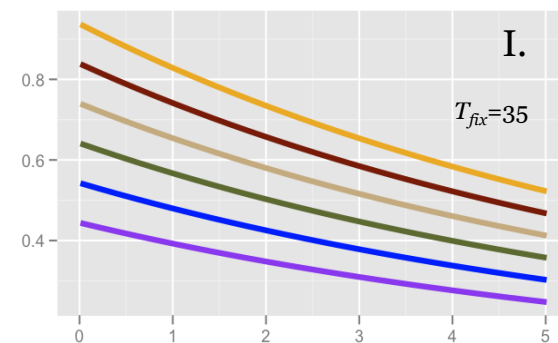

Recombination Rate (cM)

Supplement: Figure S6 — Hitchhiking effects for linked loci. Plot of recombination frequency versus change in frequency of neutral allele, ΔU. Initial frequency of neutral allele, U0, set at 0.05, and the maximum ΔU is 0.95. Population size, N, is assumed to be constant and set at 1,000. Initial frequency of selected locus, p0, varies with columns (1/2N = 0.0005, 0.05, 0.1) and strength of selection, s, varies with row (0.05, 0.1, 0.25). Tfix is number of generations at which p>0.99. (A, G, H) Simulations of a hard selective sweep assume new mutation, p, initially resides on the same haplotype as a linked allele, u. Overlapping lines demonstrates that our numerical model (orange) matches Barton's analytical solution (green) (equation 1 in [38]). (B, C, E, F, H, I) Soft sweep simulations assume p0 is 0.05 or 0.10. Different colored lines denote simulations initiated with different values of u_p, the frequency of the u allele on the p haplotype. orange u_p = 1.0; dark red u_p = 0.9; tan u_p = 0.8; dark green u_p = 0.7; blue u_p = 0.6; purple u_p = 0.5. (PDF) [file pbio.1001497.s006.pdf]
