# Supplementary material for: Indirect Evolution of Hybrid Lethality Due to Linkage with Selected Locus in Mimulus guttatus
Source: PLoS Biol. 2013 Feb 26;11(2):e1001497. doi: 10.1371/journal.pbio.1001497 (PMC3582499; doi:10.1371/journal.pbio.1001497)

Nec1 Mapping Crosses

Test Crosses-

Recombinant x Cerig10 (displayed in Figure 2C)

$F1_{BC7}$ (Tt) X Cerig10

$F1_{BC7}$ (tt) X Cerig10

Control Crosses-

Copperopolis x Cerig

Cop (TT) X Cerig10

Cop (TT) X Cerig35

NonRecombinant x Cerig10

$F1_{BC7}$ (Tt) X Cerig10

$F1_{BC7}$ (tt) X Cerig10

(also displayed in Figure 2C)

Recombinant x Cerig35

$F1_{BC7}$ (Tt) X Cerig35

$F1_{BC7}$ (tt) X Cerig35

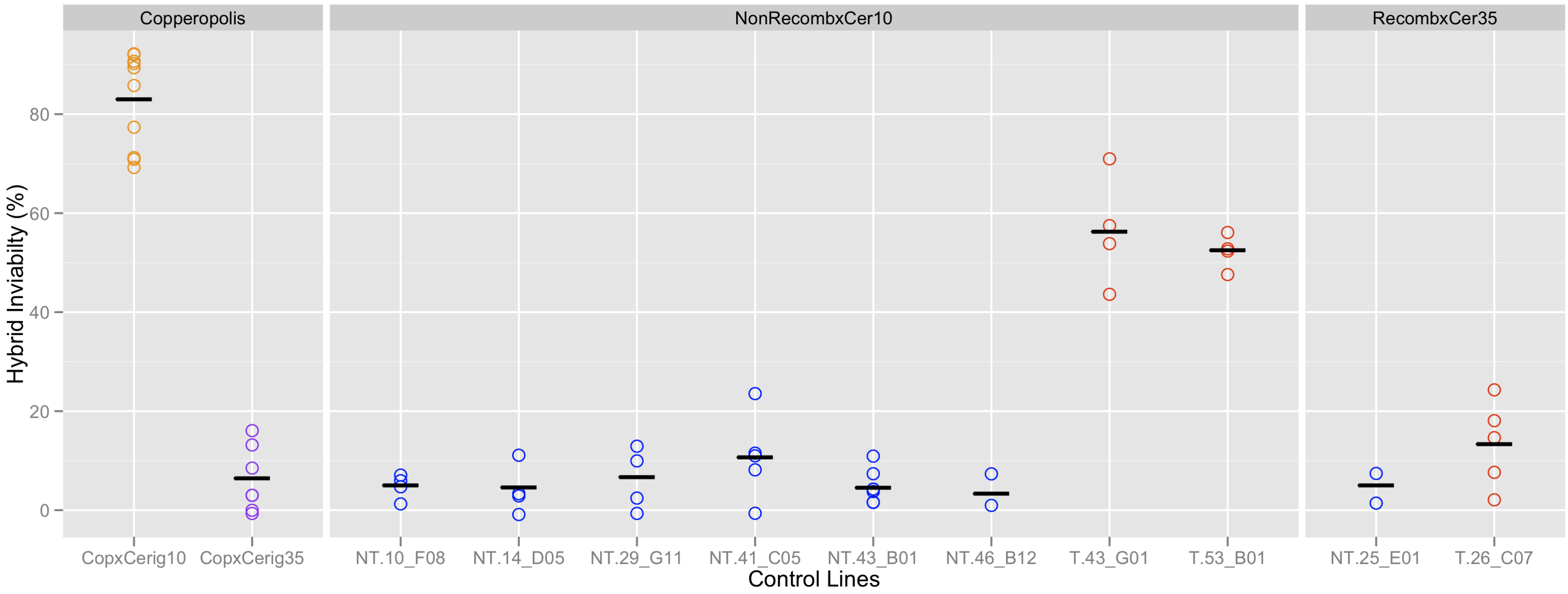

Supplement: Figure S2 — Nec1 mapping crosses. Control and test crosses for mapping Nec1. Each individual point is the frequency of lethal offspring in a block of 60; each bar is the mean for replicate blocks within a cross. The CopxCerig control crosses were conducted with five different Copperopolis genotypes and two Cerig genotypes. We observed no significant difference between individual Copperopolis genotypes; thus, we present pooled data from these crosses. The second set of control crosses was between Cerig10 or Cerig35 and control tolerant and nontolerant F1BC7 lines that have no evidence of a recombination event near Tol1. (PDF) [file pbio.1001497.s002.pdf]
