# Supplementary material for: Indirect Evolution of Hybrid Lethality Due to Linkage with Selected Locus in Mimulus guttatus
Source: PLoS Biol. 2013 Feb 26;11(2):e1001497. doi: 10.1371/journal.pbio.1001497 (PMC3582499; doi:10.1371/journal.pbio.1001497)
Supplement: Table S1 — Variation in level of hybrid inviability for lines 25E01 and 25E11. We tested for differences in the level of hybrid inviabilty between 25E01 or 25E11 and all other lines using the nonparametric Wilcoxon/Kruskal–Wallis test because the data are not normally distributed. (DOCX) [file pbio.1001497.s007.docx]

Supplemental Table 1 –

|  |  |  |  |  |  |  |
| --- | --- | --- | --- | --- | --- | --- |
| **Focal Line** | **Line** | **Control**  **Cross** | **Tolerance**  **Phenotype** | **Mean Inviability** | **Z** | **p value** |
| 25_E01 | 10_F08 | Y | NT | 5.00 | 2.467 | 0.0136 |
|  | 14_D05 | Y | NT | 4.58 | 2.467 | 0.0136 |
| Tolerance  Phenotype= | 29_G11 | Y | NT | 6.67 | -2.459 | 0.0139 |
| NT | 41_C05 | Y | NT | 10.67 | -2.470 | 0.0135 |
|  | 43_B01 | Y | NT | 5.00 | -2.941 | 0.0033 |
| Mean Inviability= | 46_B12 | Y | NT | 3.33 | -1.844 | 0.0651 |
| 38.33 | 43_G01 | Y | T | 56.25 | 1.818 | 0.0691 |
|  | 53_B01 | Y | T | 52.50 | 1.604 | 0.1087 |
|  | 19_C03 | N | NT | 8.75 | 2.459 | 0.0139 |
|  | 19_F06 | N | NT | 6.33 | 2.659 | 0.0078 |
|  | 19_H12b | N | NT | 6.67 | 2.213 | 0.0269 |
|  | 24_D09 | N | NT | 7.33 | 2.659 | 0.0078 |
|  | 32_H09 | N | NT | 6.39 | -2.817 | 0.0048 |
|  | 35_A12 | N | NT | 7.22 | -2.817 | 0.0048 |
|  | 35_B06 | N | NT | 15.00 | -1.509 | 0.1313 |
|  | 48_B01 | N | NT | 7.08 | -2.459 | 0.0139 |
|  | 25_A09 | N | T | 61.67 | -1.818 | 0.0691 |
|  | 25_E11 | N | T | 13.61 | -2.651 | 0.008 |
|  | 26_C07 | N | T | 44.58 | 0.841 | 0.4003 |
|  | 26_H02 | N | T | 50.42 | 1.103 | 0.2698 |
|  | 28_G06 | N | T | 44.58 | 0.971 | 0.3314 |
|  | 32_G02 | N | T | 61.04 | 2.331 | 0.0197 |
|  | 34_A03 | N | T | 50.83 | 1.228 | 0.2195 |
|  | 35_F09 | N | T | 56.67 | 1.818 | 0.0691 |
|  | 43_H01 | N | T | 52.92 | 1.721 | 0.0852 |
|  |  |  |  |  |  |  |
| 25_E11 | 10_F08 | Y | NT | 5.00 | 2.270 | 0.023 |
|  | 14_D05 | Y | NT | 4.58 | 1.936 | 0.0528 |
| Tolerance  Phenotype= | 29_G11 | Y | NT | 6.67 | -1.299 | 0.1939 |
| T | 41_C05 | Y | NT | 10.67 | -0.827 | 0.4081 |
| Mean Inviability= | 43_B01 | Y | NT | 5.00 | -2.661 | 0.0078 |
| 13.61 | 46_B12 | Y | NT | 3.33 | -1.687 | 0.0916 |
|  | 43_G01 | Y | T | 56.25 | 2.459 | 0.0139 |
|  | 53_B01 | Y | T | 52.50 | 2.459 | 0.0139 |
|  | 19_C03 | N | NT | 8.75 | 0.433 | 0.665 |
|  | 19_F06 | N | NT | 6.33 | 1.471 | 0.1414 |
|  | 19_H12b | N | NT | 6.67 | 1.830 | 0.0672 |
|  | 24_D09 | N | NT | 7.33 | 1.481 | 0.1386 |
|  | 25_E01 | N | NT | 38.33 | -2.651 | 0.008 |
|  | 32_H09 | N | NT | 6.39 | -1.945 | 0.0517 |
|  | 35_A12 | N | NT | 7.22 | -1.145 | 0.2521 |
|  | 35_B06 | N | NT | 15.00 | 0.000 | 1 |
|  | 48_B01 | N | NT | 7.08 | -1.299 | 0.1939 |
|  | 25_A09 | N | T | 61.67 | -2.459 | 0.0139 |
|  | 26_C07 | N | T | 44.58 | 3.041 | 0.0024 |
|  | 26_H02 | N | T | 50.42 | 2.989 | 0.0028 |
|  | 28_G06 | N | T | 44.58 | 2.459 | 0.0139 |
|  | 32_G02 | N | T | 61.04 | 3.041 | 0.0024 |
|  | 34_A03 | N | T | 50.83 | 2.908 | 0.0036 |
|  | 35_F09 | N | T | 56.67 | 2.459 | 0.0139 |
|  | 43_H01 | N | T | 52.92 | 2.467 | 0.0136 |
