# Supplementary material for: Indirect Evolution of Hybrid Lethality Due to Linkage with Selected Locus in Mimulus guttatus
Source: PLoS Biol. 2013 Feb 26;11(2):e1001497. doi: 10.1371/journal.pbio.1001497 (PMC3582499; doi:10.1371/journal.pbio.1001497)
Supplement: Table S2 — Reciprocal hybrid inviability. The observed and expected number of inviable offspring from reciprocal crosses between Cerig and Tol1 recombinant plants. ** p<0.01. (DOCX) [file pbio.1001497.s008.docx]

Supplemental Table 2

|  |  |  |  |  |  |
| --- | --- | --- | --- | --- | --- |
| Tolerance Phenotype | Pollen Donor | Total Observations | Observed Lethal | Expected Lethal | G Stat |
| NT | Cerig | 960 | 108 | 115.4 | 0.11 |
|  | Cop. | 1410 | 177 | 169.5 |  |
| T | Cerig | 1650 | 856 | 797.5 | 8.50** |
|  | Cop | 1470 | 652 | 710.5 |  |
