# Supplementary material for: Indirect Evolution of Hybrid Lethality Due to Linkage with Selected Locus in Mimulus guttatus
Source: PLoS Biol. 2013 Feb 26;11(2):e1001497. doi: 10.1371/journal.pbio.1001497 (PMC3582499; doi:10.1371/journal.pbio.1001497)
Supplement: Table S3 — Pairwise population differentiation. Pairwise population estimates of Fst and He values for Tol1UnLink markers. * p<0.025. (DOCX) [file pbio.1001497.s009.docx]

Supplemental Table 3

|  |  |  |  |  |  |  |  |  |
| --- | --- | --- | --- | --- | --- | --- | --- | --- |
|  | CCC v. OBR | |  | CCC v. HNT | |  | HNT v. OBR | |
| Locus | H_e_ | F_st_ |  | H_e_ | F_st_ |  | H_e_ | F_st_ |
| 332 | 0.207 | 0.055 |  | 0.358 | 0.036 |  | 0.287 | 0.078 |
| 836 | 0.922 | 0.054 |  | 0.936 | 0.089 |  | 0.904 | 0.045 |
| 571 | 0.865 | 0.035 |  | 0.868 | 0.086 |  | 0.871 | 0.089 |
| 617 | 0.897 | 0.015 |  | 0.907 | 0.012 |  | 0.912 | 0.021 |
| 648 | 0.921 | 0.033 |  | 0.874 | 0.068 |  | 0.880 | 0.048 |
| 278 | 0.949 | 0.050 |  | 0.949 | 0.058 |  | 0.957 | 0.053 |
| 423 | 0.939 | 0.028 |  | 0.960 | 0.073 |  | 0.978 | 0.087 |
| 672 | 0.950 | 0.183* |  | 0.839 | 0.042 |  | 0.946 | 0.159* |
| 837 | 0.742 | 0.055 |  | 0.892 | 0.190 |  | 0.886 | 0.082 |
| 641 | 0.967 | 0.073 |  | 0.977 | 0.112 |  | 0.946 | 0.033 |
| a217 | 0.782 | 0.054 |  | 0.763 | 0.114 |  | 0.705 | 0.041 |
|  |  |  |  |  |  |  |  |  |
| Mean (SD) |  | 0.058 (0.044) | |  | 0.080 (0.048) | |  | 0.067 (0.038) |
