# Supplementary material for: Indirect Evolution of Hybrid Lethality Due to Linkage with Selected Locus in Mimulus guttatus
Source: PLoS Biol. 2013 Feb 26;11(2):e1001497. doi: 10.1371/journal.pbio.1001497 (PMC3582499; doi:10.1371/journal.pbio.1001497)
Supplement: Text S1 — We provide details on genotyping the mapping population, identification of recombinant lines, and attempts to map additional scaffolds from the M. guttatus genome assembly to the Tol1 genomic region. Second, we provide additional information on our model of genetic hitchhiking under scenarios of a hard or soft selective sweep. (DOCX) [file pbio.1001497.s010.docx]

**Supplemental Text**

***Identifying Recombinant Lines***

Our mapping data only included the 36 plants confirmed to be recombinants through repeated genotyping and phenotyping. We have identified another 18 potential recombinant plants, ( 9_E06, 9_E07, 9_F06, 12_B11, 13_B06, 14_E08, 15_B05, 15_F11, 15_G01, 17_B06, 18_E03, 30_A09, 32_G08, 33_A12, 37_A08, 38_H09, 57_F05, 57_F06 ) but these lines perished in the greenhouse before we could re-test them for copper tolerance to confirm their phenotype.

One line, 19_H12, had different genotypes for tissue collected in 2007, when phenotyped for copper tolerance, and tissue collected in 2009, when phenotyped for hybrid lethality. We believe this line was contaminated while being maintained in greenhouse between 2007 and 2009, which may mean this line does not represent an independent recombination event in mapping Nec1. However, the genotypes for this line are still informative for mapping each trait and they are included in our analysis as 19_H12a and 19_H12b.

***Testing Candidate Scaffolds***

We attempted to identify additional scaffolds that map to the 0.32 cM interval between our *Sc84_37kb* marker and *Tol1* by designing markers in candidate scaffolds and testing for linkage to *Tol1* in our recombinant plants. Candidate scaffolds were defined by evidence of linkage to scaffold 84 in a recombinant inbred line mapping (RIL) population [53]. We mapped the genomic location of scaffolds, as part of the *M. guttatus* genome project, by resequencing 60 plants from our RIL population [Uffe Hellsten, JGI, personal communication]. We designed and tested 23 markers in the following seven scaffolds: **63b** (273kb, 813kb, 1.08Mb, 1.15Mb, 1.18Mb), **97c** (880kb, 907kb, 997kb), **103** (299kb, 317kb, 346kb), **157** (146kb, 300kb, 338kb, 508kb, 550kb), **238** (59kb, 160kb, 232kb, 249kb, 290kb) **460** (52kb) and **925** (8.8kb). We determined that markers *sc97c_880kb,* *sc238_59kb, sc238_232kb, sc238_249kb,* *sc157_300kb, sc157_508kb* gave reliable amplification and posses informative SNPs that distinguish tolerant and nontolerant control lines. This demonstrates these markers are located in the *Tol1* introgression region, however, genotyping data in multiple (N=6-9) recombinant lines demonstrated that that these markers are not located in between *sc84_37kb* and *Tol1* markers, nor are they located in the genomic region flanking *Tol1* (data not shown). We did not determine the exact location of these markers relative to other scaffolds in the region because we did not screen all of the recombinant lines. We have only a limited amount of DNA from these plants and once we determined that they did not map to our interval of interest, we did not genotype any additional individuals. We identified three additional markers (*sc63_1.18M, sc460_52kb, sc925_8.8kb*) that consistently amplified and contained reliable SNP variants, however none of these SNPs segregated with the tolerance phenotype in control lines. These results suggest these markers are located outside of the introgressed *Tol1* region. All other markers failed to amplify consistently. We screened marker *sc103_346kb* to determine whether it is linked to our first scaffold 103 marker, *MgSTS242* located at 749kb, or whether this scaffold is fragmented, as indicated in the RIL mapping data. We found that *sc103_346kb* segregates with *MgSTS242* suggesting this scaffold is contiguous in the Copperopolis genome.

**Hitchhiking Model**

To simulate hitchhiking on tightly linked sites following a hard or soft selective sweep, we use the two-locus model of genetic hitchhiking described by Maynard Smith and Haigh [35] and Barton [38]. We follow Barton’s notation for the variables in our simulations. This model assumes that selection acts on a single locus, with alleles *p* and *q*. Selection is deterministic and genotypic fitness is additive: *PP* = 1 +2s; *PQ* = 1+s *QQ* = 1. The populations are assumed to be in Hardy Weinberg Equilibrium and the change in allele frequency of *p* for the next generation is: *p'* = [*p*^2^(1+2s) + *pq*(1+s)] / [*p*^2^(1+2s) + 2*pq*(1+s) + *q*^2^(1)]. A neutral locus, with alleles *u* and *v,* is located *r* Morgans from the selected locus*.* The two focal variables in this model are the frequency of the *u* allele on the *p* haplotype, *u_p_*, and on the *q* haplotype, *u_q_*. In the hard sweep model, the initial value of *u_p_* = 1, because there is only a single copy of *p* allele. In the soft sweep model, we conduct independent simulations with the initial values of *u_p_* varying from 1.0, complete association, to 0.5, weak association. The frequency of *u* is calculated as *u* = *u_p_*•*p* + *u_q_*•*q* [38, page 1554]. Each generation, the new values of *u_p_*  and *u_q_* are calculated according to equations: *u_p_* ' = *rq*(*u_q_* - *u_p_*) and *u_q_'* = *rp*(*u_p_* - *u_q_*) [38, page 1554].

To explore the effects of selection on the allele frequency of a linked neutral allele, we conduct simulations using these equations to calculate the change in frequency of *u* when selection acts on the tightly linked allele, *p.* The simulations are run under a wide range of initial conditions, which are described in the legend of Supplemental Figure 6. The simulations end once the *p* has reached a frequency of >0.99. The source code, written in C, is deposited here: http://openwetware.org/images/9/99/Hitchhiking_model.c.
